# Diabetes MILES--Australia (management and impact for long-term empowerment and success): methods and sample characteristics of a national survey of the psychological aspects of living with type 1 or type 2 diabetes in Australian adults

**DOI:** 10.1186/1471-2458-12-120

**Published:** 2012-02-12

**Authors:** Jane Speight, Jessica L Browne, Elizabeth Holmes-Truscott, Christel Hendrieckx, Frans Pouwer

**Affiliations:** 1 The Australian Centre for Behavioural Research in Diabetes, Diabetes Australia--Vic, 570 Elizabeth Street, Melbourne, VIC 3000, Australia; 2 Centre for Mental Health and Wellbeing Research, School of Psychology, Deakin University, Burwood, VIC 3125, Australia; 3 AHP Research, Hornchurch, Essex, UK; 4 Center of Research on Psychology in Somatic diseases (CoRPS) FSW, Tilburg University, Warandelaan 2, 5037 AB Tilburg, The Netherlands

**Keywords:** Diabetes, Psychology, National survey, Self-care, Quality of life, Diabetes-related distress, Depression

## Abstract

**Background:**

Successful management of diabetes requires attention to the behavioural, psychological and social aspects of this progressive condition. The Diabetes MILES (Management and Impact for Long-term Empowerment and Success) Study is an international collaborative. Diabetes MILES--Australia, the first Diabetes MILES initiative to be undertaken, was a national survey of adults living with type 1 or type 2 diabetes in Australia. The aim of this study was to gather data that will provide insights into how Australians manage their diabetes, the support they receive and the impact of diabetes on their lives, as well as to use the data to validate new diabetes outcome measures.

**Methods/design:**

The survey was designed to include a core set of self-report measures, as well as modules specific to diabetes type or management regimens. Other measures or items were included in only half of the surveys. Cognitive debriefing interviews with 20 participants ensured the survey content was relevant and easily understood. In July 2011, the survey was posted to 15,000 adults (aged 18-70 years) with type 1 or type 2 diabetes selected randomly from the National Diabetes Services Scheme (NDSS) database. An online version of the survey was advertised nationally. A total of 3,338 eligible Australians took part; most (70.4%) completed the postal survey. Respondents of both diabetes types and genders, and of all ages, were adequately represented in both the postal and online survey sub-samples. More people with type 2 diabetes than type 1 diabetes took part in Diabetes MILES--Australia (58.8% versus 41.2%). Most respondents spoke English as their main language, were married/in a de facto relationship, had at least a high school education, were occupied in paid work, had an annual household income > $AUS40,000, and lived in metropolitan areas.

**Discussion:**

A potential limitation of the study is the under-representation of respondents from culturally and linguistically diverse backgrounds (including Aboriginal and Torres Strait Islander origin). Diabetes MILES--Australia represents a major achievement in the study of diabetes in Australia, where for the first time, the focus is on psychosocial and behavioural aspects of this condition at a national level.

## Background

Diabetes is a common and progressive long-term condition, which places a significant burden of self-management on the individual. The prevalence of all types of diabetes is increasing, with type 2 diabetes growing at epidemic proportions [1]. The total number of people with diabetes worldwide was conservatively estimated to increase from 171 million in 2000 to 366 million in 2030 [2].

Self-management of diabetes (in all its forms) is a complex behavioural and social process, requiring not only a comprehensive understanding of the condition but also high levels of self-efficacy, perceived control and empowerment [3]. Unsurprisingly, having diabetes can negatively impact the quality of life of people living with the condition [4,5]. A considerable number of studies have found relatively high levels of distress, and, in a substantial minority, significant depressive symptomatology [6]. Furthermore, the onset of complications (such as retinopathy, neuropathy, kidney damage, heart disease and stroke) can exacerbate the psychological impact of this progressive condition [7]. Thus, the association between diabetes and psychological burden appears to be a vicious cycle and we need to identify ways to reduce this burden. In addition to understanding the psychological health and well-being of people with diabetes, research is needed to establish a greater understanding of perceptions and misconceptions among people with diabetes, their health beliefs and the personal values which inform their self-management.

Such research has been concentrated for the most part in the US and Europe, with very little being conducted in Australia. The Diabetes Attitudes Wishes and Needs (DAWN) Study is probably the only large-scale study that has focused on the psychological impact of diabetes and the implications for improvements to health care for people with diabetes [8]. This cross-national study, which included an Australian sample, highlighted the importance of a positive and collaborative relationship between health care providers and people with diabetes [9], as well as the relatively high prevalence of diabetes-related distress amongst people with diabetes [10], and the role of psychological and social barriers to self-care and medication initiation and management [11,12]. The DAWN Study highlighted important avenues for future research into the psychological aspects of living with diabetes that need to be pursued in Australia. DAWN 2, a related study with increased breadth, is currently underway but Australia is not included in this new initiative.

The Living With Diabetes Study is a population-based cohort study of the quality of life and well-being of people with diabetes living in Queensland, Australia [13]. One of the primary aims of this study is to evaluate a state-based strategy for the management of chronic conditions, and thus the Living With Diabetes Study has been undertaken in one Australian state only.

The Diabetes MILES Study has been established as an international collaborative, the aim of which is to conduct a series of national surveys of people with type 1 or type 2 diabetes in various countries. Diabetes MILES--Australia is the first Diabetes MILES initiative, and was conceived to provide a national survey of the psychological health, self-care beliefs and activities, and unmet needs of Australians living with type 1 or type 2 diabetes. The Diabetes MILES--Australia 2011 survey was built on four central themes: management, impact, empowerment, and success.

### Survey themes

#### Management

The burden of the management of diabetes falls largely to the individual living with the condition. Self-management, or self-care, activities are crucial for achieving and maintaining optimal blood glucose levels, and preventing diabetes-related complications. Self-care is defined as 'activities that individuals, families and communities undertake with the intention of enhancing health, preventing disease, limiting illness and restoring health. They are undertaken by lay people on their own behalf, either separately or in participative collaboration with health professionals' [14]. Diabetes self-care can involve dietary modifications, taking medications as recommended, regular physical activity, foot care and self-monitoring of blood glucose.

Self-care requires not only a comprehensive understanding of diabetes but also belief in one's capabilities, motivation to perform complex self-care activities, and a supportive environment (of family, friends and health care professionals). Self-care is central to both the experience of living with diabetes and the optimisation of diabetes outcomes, and thus ongoing research in this area is essential.

#### Impact

International reviews have found clinical levels of depression in up to 12% of people with type 1 diabetes and 18% of people with type 2 [15,16]. Many more are likely to report some level of diabetes-related distress [6] and, on average, diabetes has a negative impact on quality of life [5]. Understanding the psychological, emotional, and social impact of diabetes is essential for the development and evaluation of new therapies and interventions, including but not limited to psychological therapies. For example, identifying the negative impact of diabetes on dietary freedom [5] was instrumental in hypothesising that the Dose Adjustment For Normal Eating (DAFNE) program [17] would offer substantial benefits for quality of life, benefits which have been well-maintained for up to four years in combination with partially maintained improvements in glycaemic levels [18]. We need to advance our understanding of the psychological, emotional, and social impact of diabetes so that care and support for people living with the condition can be optimised.

#### Empowerment

A person with diabetes who is empowered has high perceived self-efficacy, is actively engaged in their own health care (including self-care), and seeks out necessary support and information. Empowerment is undoubtedly philosophically desirable, and importantly, evidence suggests that it is the only way to achieve optimal, sustainable long-term outcomes [19]. Unfortunately, it can be rare in everyday clinical practice, where both the health care professional and person with diabetes often adopt traditional roles, which can result in the person with diabetes becoming a passive recipient of health care and feeling de-motivated. Access to diabetes education and utilisation of healthcare resources are examples of the means through which people with diabetes can be empowered to best manage their condition, and both are critical in achieving optimal biomedical outcomes in diabetes. Education and support programs are known to enhance self-management and improve psychosocial outcomes but are not widely available. Figures from the UK national diabetes audit [20] indicate that a large proportion of people with diabetes are not undergoing the regular checks recommended for careful monitoring of their condition. It is important to understand the role of empowerment in successful diabetes management and utilisation of services, and to subsequently promote and facilitate patient empowerment in health care and other settings.

#### Success

In addition to understanding the psychological health and well-being of Australians with diabetes, research is needed to develop a greater understanding of the role of health beliefs, and the personal values which inform self-management and willingness to engage with treatments as recommended by health care professionals. Health beliefs (e.g. perceived severity of disease, perceived efficacy of treatment) and aspects of positive mental health (such as resilience, self-esteem, optimism) are important mediators of diabetes outcomes [21-23]. Hardiness, is associated with lower distress; low rates of complications and is a good predictor of optimal outcomes, independent of education [22]. Social and peer support is also implicated in better outcomes [24,25]. It is important to further examine the role of a person's disposition, and their social context, in contributing to living successfully with diabetes [26].

### Aims

The primary aims of Diabetes MILES--Australia are to:

• conduct a large-scale survey of Australian adults with type 1 or type 2 diabetes with a focus on exploring the psychological, behavioural and social factors relevant to living with diabetes

• ensure the suitability of the survey as the baseline for a longitudinal (repeated cross-sectional and cohort) study

• use the results to raise awareness of the psychological health and unmet needs of Australian adults living with diabetes.

Primary research questions to be addressed by Diabetes MILES--Australia include (but are not limited to):

• What characterises living successfully with diabetes? (e.g. long diabetes duration with minimal physical complications and optimal psychological health)

• What is the prevalence of impaired awareness of hypoglycaemia and severe hypoglycaemia among people with type 1 diabetes?

• What are unmet needs of Australian adults living with diabetes?

• What is the impact of diabetes on the well-being of Australians living with the condition?

• Is the Quality of Life Questionnaire--Diabetes [27] a valid and reliable measure of the impact of diabetes on quality of life?

• Is the Diabetes Self-Care Inventory--Revised a valid and reliable measure of self-care beliefs, attitudes, and behaviours?

• Are postal and internet-based surveys equally suitable methods for conducting a national survey?

• How do two measures of diabetes-related distress (the Diabetes Distress Scale (DDS)[28] and the Problem Areas In Diabetes questionnaire (PAID)[29]) compare in terms of psychometric properties and utility?

• What is the prevalence of clinically significant depression, anxiety and diabetes-related distress in Australian adults living with diabetes?

## Methods/design

### Ethics approval

Ethics approval was granted by Deakin University Human Research Ethics Committee (reference number 2011-046). A plain language statement and consent form were developed for the cognitive debriefing phase as well as for the main national survey.

### Establishment of the reference group

A multidisciplinary reference group was established comprising 32 representatives from psychology, nursing, population health, health economics, pharmacy, endocrinology, and general practice (see http://www.diabetesMILES.org). Most reference group members are Australian but eight international academics are also involved. The role of the reference group was to advise on survey content (concepts, measures and individual variables) and research questions. An important ongoing role for the reference group is to collaborate on dissemination of the survey results.

### Phase 1: Selection of psychological measures

The Diabetes MILES--Australia 2011 survey was developed through the following process: initial measure selection occurred after phases of measure identification, assessment and consultation, cognitive debriefing of standardised measures and survey-specific items before survey distribution.

#### Identification

Within each study theme (management, impact, empowerment and success) a number of relevant concepts were identified. For each concept a questionnaire search was completed. Measures were considered for use only if they were appropriate for use with adults (aged 18-70 years) and were designed for any of the following population types: generic, diabetes specific, chronic condition specific.

#### Assessment

For each measure the following information was collected and assessed: length, reliability and validity, previous use in Australia and internationally, and use within diabetes-specific populations.

A number of important concepts of the study were not adequately or succinctly measured through already established questionnaires, necessitating the drafting of a number of items by the research team at this point. It became clear that several measures had not been validated for use in Australia but one of the pivotal roles of Diabetes MILES--Australia is to provide the dataset upon which psychometric validation can be conducted. Linguistic validation (i.e. the extent to which measures were suitable for use in English for Australia) was considered during selection by the research team (two of whom are Australian) and through cognitive debriefing (see below) with Australians living with diabetes.

#### Consultation

After the initial assessment had been made, an item-bank was compiled for consideration by the wider reference group who were asked to contribute detailed feedback and suggestions for further development of the item-bank. The item-bank was circulated for consultation three times. On each occasion the feedback received either confirmed the use of an identified measure or led to identification and assessment of other measures. The Principal Investigator (JS) and the research team made all final decisions regarding the survey content.

### Phase 2: Pilot study and cognitive debriefing

The aim of the pilot study was to ensure that the survey content was relevant and suitable for Australian adults with type 1 or type 2 diabetes. This involved asking participants to complete the survey and then take part in a cognitive debriefing (CD) interview.

#### Recruitment

Participants were recruited through advertisements in relevant diabetes media (Diabetes Australia-Vic magazines, websites and e-newsletters) in the state of Victoria. Potential participants contacted the research team by telephone or email and they were sent (by email, post or fax) a copy of the plain language statement, and a consent form to sign. Participants were eligible if they met the inclusion criteria for the larger national survey sample (see below). Decisions regarding recruitment were based on responses to a brief set of screening questions. This ensured a representative sample, relevant to the aims of this phase of the study (i.e. an adequate mix of men/women, ages, diabetes types and treatment, education levels and occupations). In addition, participants needed to be willing to attend interviews held in Melbourne city centre, Burwood (suburb of Melbourne) or Geelong (regional city in Victoria, although no participants chose this option) to minimise travel expenses and to enable this phase of the project to progress quickly.

#### Procedure

Ahead of the CD session, participants were posted a copy of the survey booklet plus a letter requesting that they read and/or complete the survey booklet no more than one day before the interview. Participants were invited to make note of any survey instructions, sections, questions or response options which were unclear or of concern to them. Upon arrival at the CD session, participants were asked to provide their consent form and were reminded of their participatory rights. All interviews were audio recorded in a de-identified digital format. Participants were free to refuse to answer any of the questions.

Participants were first asked to comment on the survey as a whole in terms of relevance, readability, acceptability, language and length. Participants were also asked how long it took them to complete the survey and the extent to which they found it easy or difficult to complete. Participants were then asked a number of similar questions about each section of the survey, a selection of specific standardised psychological measures, and study-specific items.

#### Results

CD was conducted with 20 adults living in and around Melbourne: 12 participants with type 1 diabetes (seven women; mean age = 43.5 ± 9.6 years (range: 25-56 years)); eight with type 2 diabetes (seven women; mean age = 55.9 ± 15.2 years (range: 21-64 years)). The education level of participants ranged from secondary school to higher university degree.

Participants indicated that it took a mean of 58 min to complete or read through the survey (range: 20-120 min). The CD interviews had a mean duration of 51 min (range: 20-109 min).

Some minor changes to the survey were made in response to feedback received, such as improvements to the survey booklet layout, amending instructions so they were simpler and more consistent, and a small number of measures were excluded from the survey to reduce its length.

### Phase 3: Finalising survey content and study materials

All survey modifications made as a result of the CD sessions were approved by the Deakin University Human Research Ethics Committee.

At the completion of this process the detailed concept and measure index (Table 1) was finalised. This index lists all concepts investigated in the Diabetes MILES--Australia 2011 survey, the measures used to assess these concepts, as well as the survey version in which the measure was included. Minimising respondent burden and maximising relevance (e.g. to diabetes type/treatment) were crucial considerations. Thus, the item bank was split into six surveys: an 'A' and 'B' version for each participant group (type 1 diabetes (T1), type 2 diabetes insulin-treated (T2I), type 2 diabetes non-insulin-treated (T2)). This enabled inclusion of some questionnaires which may have otherwise been excluded due to space limitations. Core measures/items were included in all survey versions, while others were included in only one or two versions (details in Table 1). Completion of the survey was expected to take approximately one hour.

**Table 1:** Concepts, measures and variables included in the Diabetes MILES--Australia 2011 Survey

Concept	Measure or variable	Survey version
*Part 1: Your feelings In general*		

Subjective Wellbeing	PWI: Personal Wellbeing Index [30]	ALL

Emotional Wellbeing	WHO-5: World Health Organisation Well-being index [31]	ALL

Depression	PHQ-9: Patient Health Questionnaire [32]^	ALL

Anxiety	GAD-7: General Anxiety Disorder Questionnaire [33]^	ALL

*Part 2: Your feelings about diabetes*		

Diabetes specific quality of life	QoL-Q Diabetes [27]	ALL

Diabetes-specific distress	DDS: Diabetes Distress Screening Scale [28]^#^; PAID: Problem Areas In Diabetes scale [29]^#^^	ALL

Diabetes-specific positive well-being	4-item subscale of the W-BQ28: Wellbeing Questionnaire [34]	ALL

*Part 3: Your general health*		

General health	EQ5D-5L [35]; 3 items (general health in past 4 weeks; change in health in past year)*	ALL

Miscellaneous	7 items: other conditions/co-morbidities*^; sleep*; transplants*; dialysis*; weight loss surgery*	ALL

*Part 4: Support from health professionals*		

Health consultations	29 items (main health care professionals, access, reliance, distance, consistency, continuity, distance/cost as an obstacle, timely appointment attendance)*^ inspired by the ALSHW Survey 2009	ALL

Healthcare and self-management	RSSM: Resources and Support for chronic illness Self-Management scale [36]	T1A, T2IA, T2A

Structured education	4 items* inspired by the IDF Diabetes Atlas 2009	T1A, T2IA, T2A

Empowerment	DES-SF: Diabetes Empowerment Scale Short Form [37]	ALL

Health literacy	HeLMS: Health Literacy Management Scale (seven of eight subscales included)	ALL

*Part 5: Support from friends and family*		

Peer/family support	3 items* inspired by Tang et al, 2008 [38]; DFSC: Diabetes Family Support and Conflict [39], Diabetes support group involvement*	T1B, T2IB, T2B

*Part 6: Your diabetes*		

Diabetes history	Age at diagnosis*^; diabetes type*^; current treatment regimen*^	ALL

Blood glucose monitoring and recording	6 items (satisfaction, monitoring, recording, action)* inspired by the DAFNE self-management questionnaire (in development)*	ALL

Self-care behaviours & attitudes	Diabetes Self-Care Inventory--Revised^^ ^adapted to include smoking items* inspired by the Smoking and Health Survey 2010 (the Cancer Council Vic)[40]	ALL

Medication adherence	MARS: Medication Adherence Rating Scales [41] (Insulin and/or Medicine)	ALL

Blood glucose level and HbA1_C _targets	4 items*^	T1A, T2IA, T2A

Hypoglycaemia awareness	The Gold Score [42]; 5 items from the HypoA-Q: Hypo Awareness Questionnaire [43]^	T1A

Hypoglycaemia treatment	1 item*	T1A

Fear of hypoglycaemia	2 items* inspired by the Hypoglycaemia Fear Scale [44]	T1A

Insulin restriction	Items inspired by Diabetes Australia-Vic's Type 1 Diabetes and Eating Disorders Online Survey 2008 Report*	T1B

Disordered eating	6 items* inspired by Type 1 Diabetes and Eating Disorders Online Survey 2008 report	T1B

Perceived behavioural control	15 items*	T2IB, T2B

Physical activity	IPAQ: International Physical Activity Questionnaire (Short Form) [45]; 5 items*	T2IB, T2B

Psychological insulin resistance	ITAS: Insulin Treatment Appraisal Scale [46]^^^	T2IB, T2A

*Part 7: Your thoughts and beliefs*		

Optimism	LOT-R: Life Orientation Test--Revised [47]	T1B, T2IB, T2B

Perceived self-efficacy	GSE: General Self-Efficacy Scale [48]	T1B, T2IB, T2B

Self-esteem	Rosenberg Self-Esteem Scale [49]	T1B, T2IB, T2B

Beliefs about illness	BIPQ: Brief Illness Perceptions Questionnaire (diabetes version) [50]^^^	T1A, T2IA, T2A

Beliefs about medicines	BMQ: Beliefs about Medicines Questionnaire (medicine/insulin and general) [51]	T1A, T2IA, T2A

*Part 8: About you*		

Demographics	25 items (e.g. sex, age, weight/height, education, occupation, disability)*^ inspired by the ALSWH 2009	ALL

Financial hardship	Economic Hardship Questionnaire [52]; 8 items*	T1B, T2IB, T2B

NDSS access	4 items* from previous NDSS surveys	ALL

*Part 9: Have we missed anything?*		

Further comments	Free-text box	ALL

*Part 10: Future Research*		

What research would you like to see?	Free-text box	ALL

Interest in future research	2 items*	ALL

* designed by research team in the absence of relevant and suitable standardised measures

^# ^measure/item included in only 50% of surveys in order to reduce respondent burden

^^ ^Diabetes MILES Study core measures to be included in all surveys of adults living with type 1 or type 2 diabetes (e.g. Diabetes MILES--Australia, Diabetes MILES--The Netherlands)

*ALSWH *Australian Longitudinal Study on Women's Health

Survey types: T1: Type 1 diabetes; T2I: Type 2 insulin-treated diabetes; T2: Type 2 non-insulin-treated diabetes. For each type, there was an 'A' and 'B' version.

#### Preparation of postal and online surveys

Hard copy and online versions of the survey were developed. A contract research organisation (CRO) prepared the hard copy survey booklets in a format that enabled responses to be scanned for automated data entry. The online survey was designed by a web developer based in the School of Psychology, Deakin University.

The postal survey booklets (specific to diabetes type and treatment regimen of recipient; version A or B randomly assigned) were packaged with a reply-paid envelope, a plain language statement and withdrawal of consent form, contact details form and a language other than English (LOTE) form (both optional to complete). For practical reasons, the survey was not available in LOTE but survey recipients were invited to indicate if they would like to complete a similar survey in their own language in the future (if it became available). This statement was translated into nine of the most commonly spoken languages in Australia: Arabic, Cantonese, Greek, Italian, Macedonian, Mandarin, Spanish, Turkish, and Vietnamese.

### Phase 4a: Data collection--national postal survey

Approximately six weeks was allocated for data collection (postal survey distribution and return, and online completion).

#### Study population

The postal survey was distributed to a random sample of 15,000 registrants of the National Diabetes Services Scheme (NDSS). The NDSS register includes approximately one million Australians diagnosed with diabetes (e.g. type 1, type 2, gestational). NDSS registrants were eligible for selection if they met the following inclusion criteria:

• living with type 1 or type 2 diabetes

• aged 18 to 70 years

• previously indicated consent to be contacted about future research; approximately 250,000 (25%) of NDSS registrants

The sample size of 15,000 was chosen in anticipation of a 20% response rate (N = 3,000), offering a substantial dataset and enabling meaningful subgroup analyses. The sample was stratified (based on information provided on the NDSS register) to ensure adequate representation of specific types of diabetes and treatment regimens:

• Type 1 diabetes: 6,000 registrants (40% of total sample)

- 5,400 using insulin injections

- 600 using pump therapy

• Type 2 diabetes: 9,000 registrants (60% of total sample)

- 4,500 using insulin

- 4,500 not using insulin

The sample was not stratified by gender.

#### Procedure

All study materials were supplied to the Diabetes Australia--Vic mail co-ordination team who addressed and posted all surveys. The research team had no direct involvement in the random selection or in the handling of potential participant contact details.

Participants received the survey package by post and were asked to return the completed survey using the reply-paid envelope provided before 31^st ^July 2011. In practice, there were some late arrivals and we included all returns received by 31^st ^August.

### Phase 4b: Data collection--national online survey

#### Study population

The online survey (including details of the website URL) was advertised nationally through a variety of media:

• The NDSS website

• Diabetes Australia (national and state-based) websites, magazines and newsletters (including e-newsletters)

• posters advertising the survey were distributed through various healthcare professional networks for prominent placement in diabetes clinics

• newsletters/e-newsletters of a range of national and state-based health organisations, societies, membership bases (e.g. sanofi aventis, Medtronic, Type 1 Network (Reality Check))

Postal survey recipients were also offered the option to complete the survey online (rather than by paper and pen).

#### Procedure

Upon accessing the online survey, participants were encouraged to read the plain language statement and to tick a box to indicate informed consent to proceed. The online survey was identical to the postal survey except for the need to change the order of some primary questions to establish the survey version relevant for the participants, as well as the inclusion of a question asking how participants heard about the survey. Initial screening questions (e.g. age, type and treatment of diabetes and ability to communicate in English) were used to ensure that participants were eligible and to tailor the presentation of subsequent survey questions. Participants were randomly assigned to version A or B of the survey. Those who were ineligible were presented with a screen thanking them for their interest and informing them that they were ineligible to complete the rest of the survey. The eligibility criteria were identical to those used for the postal survey, except NDSS registration was not a requirement.

### Phase 5: Data handling and analyses

Completed postal surveys were received for initial processing and de-identification (in the instances where the contact details form was returned) by the research team. De-identified surveys were then forwarded to the CRO, accompanied by a data dictionary, for scanning and data entry. The web developer downloaded the data from the online survey into a spreadsheet that was also forwarded to the CRO for merging with the postal survey data. The CRO then provided the research team with a data file that contained the data from all returned surveys (postal and online), plus scanned images of each page of all postal surveys. All hard copies of the survey booklets were returned to the research team for secure storage.

Statistical analyses will be performed using the Statistical Package for the Social Sciences (SPSS) version 17.0. Given the anticipated large sample size, Bonferroni corrections will be used to apply more conservative significance levels for all analyses. Differences between subgroups will be determined using χ^2 ^tests for categorical data and independent samples t-tests or ANOVAs for continuous variables. Further analyses (e.g. principal components analysis, multiple regression) may be used dependent on specific research questions and will be reported in subsequent papers as relevant. Sample characteristics are presented below using frequencies (N (%)) for categorical variables and mean ± standard deviation for continuous variables.

### Response rates

In total, 3,833 people responded to the Diabetes MILES--Australia 2011 survey:

• 987 completed the online survey, of which 220 participants indicated that they had received the postal survey.

• 2,351 completed the postal survey or responded to the online survey after receiving the postal version, a response rate of 17%. However, 541 postal surveys were returned to sender, as the addressee was no longer at the address provided by the NDSS, or was deceased. Thus, the adjusted response rate is 18%.

Of the total 3,833 respondents, 495 did not meet all of the eligibility criteria (e.g. did not have type 1 or type 2 diabetes, aged younger than 18 or older than 70, could not complete survey in English without assistance) and were excluded from the sample. Subsequent analyses presented in this and other papers arising from Diabetes MILES--Australia refer only to the final sample of 3,338 eligible respondents. Response rates of eligible participants for the postal versus online surveys are presented in Table 2.

**Table 2:** Response rates for postal versus online survey*

State/territory	Total respondents N (%)	Respondents to postal survey N (%)	Respondents to online survey N (%)
**Total sample**	**3338 (100)**	**2351 (70.4)**	**987 (29.6)**

**Type 1 diabetes**	**1376 (41.2)**	**865 (36.8)**	**511 (51.8)**

ACT	39 (1.2)	17 (0.7)	22 (2.3)

NSW	295 (9.0)	233 (10.0)	62 (6.4)

NT	19 (0.6)	19 (0.8)	0 (0.0)

QLD	266 (8.1)	203 (8.7)	63 (6.5)

SA	68 (2.1)	53 (2.3)	15 (1.6)

TAS	30 (0.9)	21 (0.9)	9 (0.9)

VIC	497 (15.1)	200 (8.6)	297 (30.9)

WA	137 (4.2)	113 (4.8)	24 (2.5)

**Type 2 diabetes**	**1962 (58.8)**	**1486 (63.2)**	**476 (48.2)**

ACT	28 (0.8)	22 (0.9)	6 (0.6)

NSW	477 (14.5)	427 (18.3)	50 (5.2)

NT	66 (2.0)	66 (2.8)	0 (0.0)

QLD	327 (9.9)	284 (12.2)	43 (4.5)

SA	135 (4.1)	130 (5.6)	5 (0.5)

TAS	64 (1.9)	59 (2.5)	5 (0.5)

VIC	646 (19.6)	328 (14.1)	318 (33.1)

WA	199 (6.0)	156 (6.7)	43 (4.5)

**Men**	**1525 (46.2)**	**1137 (48.9)**	**387 (39.6)**

18-24 years	47 (1.4)	37 (1.6)	10 (1.0)

25-34 years	103 (3.1)	70 (3.0)	33 (3.4)

35-44 years	152 (4.6)	106 (4.6)	46 (4.7)

45-54 years	292 (8.8)	206 (8.9)	86 (8.8)

55-64 years	560 (17.0)	408 (17.6)	152 (15.5)

65-70 years	370 (11.2)	310 (13.3)	60 (6.1)

**Women**	**1778 (53.8)**	**1187 (51.1)**	**591 (60.4)**

18-24 years	126 (3.8)	94 (4.0)	32 (3.3)

25-34 years	226 (6.8)	128 (5.5)	98 (10.0)

35-44 years	259 (7.8)	150 (6.5)	109 (11.1)

45-54 years	387 (11.7)	237 (10.2)	150 (15.3)

55-64 years	502 (15.2)	355 (15.3)	147 (15.0)

65-70 years	278 (8.4)	223 (9.6)	55 (5.6)

ACT: Australian Capital Territory; NSW: New South Wales; NT: Northern Territory; QLD: Queensland; SA: South Australia; TAS: Tasmania; VIC: Victoria; WA: Western Australia

* Table refers only to eligible participants. Total N is not always consistent with total sample size due to missing data on some items

The representativeness of the sample can be determined by comparing respondents on key characteristics to NDSS registrants. More than half (58%) of the Diabetes MILES--Australia sample was from the states of New South Wales or Victoria and by comparison, 59% of NDSS registrants are from these two states^a^. Substantially smaller proportions of people from other Australian states and territories are NDSS registrants, and this was also reflected in our study sample. Nationally, 53% of NDSS registrants are men, as compared to 46% of our study sample, suggesting that women were slightly over-sampled in the Diabetes MILES--Australia study^b^.

### Sample characteristics

Respondents were asked to indicate their diabetes type as well as to provide a range of demographic information (see Table 3). People with type 2 diabetes were more highly represented in the sample than people with type 1 (58.8% versus 41.2%), proportionate to the ratio of surveys distributed (60% versus 40% respectively). Men and women were represented almost equally in the sample (46.2% versus 53.8% respectively). Most respondents spoke English as their main language, were married or in a de facto relationship, had at least a high school education, lived in metropolitan areas, were occupied in paid employment, and had an annual household income of more than $AUS40,000 (approximately $US39,600 or €30,563) per annum. Only a very small proportion (1.7%) of respondents were from an Aboriginal and/or Torres Strait Islander (ATSI) background, and only 3% of the total sample indicated that they mainly spoke a language other than English at home. However, 25.4% of respondents indicated that they were born in a country other than Australia, which is representative of the broader Australian population [53].

**Table 3:** Sample characteristics: Diabetes MILES--Australia 2011 cohort

	N (%) or Mean ± SD (Range)		
	
	Type 1	Type 2 N (%)	Total N* (%)
**TOTAL**	**1376 (41.2)**	**1962 (58.8)**	**3338 (100)**

**Gender--female**	825 (60.6)	953 (49.1)	1778 (53.8)

**Age--years**	41.98 ± 13.96 (18-70)	58.55 ± 8.71 (19-70)	51.71 ± 13.84 (18-70)

**Aboriginal or Torres Strait Islander origin**	10 (1.0)	39 (2.1)	49 (1.7)

**Main language spoken at home--English**	1322 (96.1)	1882 (95.9)	3204 (96.0)

**Country of birth--Australia**	1061 (77.8)	1412 (72.4)	2473 (74.6)

**Marital status**			

Single	257 (18.9)	193 (9.9)	450 (13.6)

Steady relationship	99 (7.3)	26 (1.3)	125 (3.8)

Married or De facto relationship	895 (65.9)	1375 (70.7)	2270 (68.7)

Separated	25 (1.8)	58 (3.0)	83 (2.5)

Divorced	68 (5.0)	187 (9.6)	255 (7.7)

Widowed	13 (1.0)	83 (4.3)	96 (2.9)

**Education**			

No qualifications	57 (4.2)	209 (10.8)	266 (8.1)

School/Intermediate Certificate	83 (6.1)	259 (13.4)	342 (10.4)

High School/Leaving Certificate	261 (19.2)	370 (19.1)	631 (19.1)

Trade/Apprenticeship	95 (7.0)	167 (8.6)	262 (7.9)

Certificate/Diploma	293 (21.5)	428 (22.1)	721 (21.6)

University degree	341 (25.1)	241 (12.4)	582 (17.7)

Higher university degree	190 (14.0)	162 (8.4)	352 (10.5)

**Occupation**			

Unemployed	86 (6.4)	151 (7.8)	237 (7.2)

Full time student	82 (6.1)	13 (0.7)	95 (2.9)

Retired	134 (9.9)	744 (38.5)	878 (26.8)

Homemaker/Carer/Volunteer	106 (7.8)	168 (8.7)	274 (8.4)

Labourer	66 (4.9)	82 (4.2)	148 (4.5)

Clerical/Sales/Service	222 (16.4)	221 (11.5)	443 (13.5)

Tradesperson	78 (5.8)	84 (4.4)	162 (4.9)

Associate professional	158 (11.7)	152 (7.9)	310 (9.4)

Professional	368 (27.2)	240 (12.4)	608 (18.5)

Director	37 (2.7)	38 (2.0)	75 (2.3)

**Annual household income ($)**			

≤ 20,000	162 (12.3)	434 (23.7)	596 (18.9)

20,001-40,000	144 (10.9)	415 (22.7)	559 (17.8)

40,001-60,000	218 (16.6)	363 (19.8)	581 (18.5)

60,001-100,000	358 (27.2)	341 (18.6)	699 (22.2)

100,001-150,000	246 (18.7)	181 (9.9)	427 (13.6)

> 150,000	187 (14.2)	93 (5.1)	280 (8.9)

**Geographical location**			

Metropolitan	793 (58.7)	907 (47.0)	1700 (51.8)

Regional	369 (27.3)	555 (28.8)	924 (28.2)

Rural	186 (13.8)	464 (24.0)	650 (19.8)

*Total N reported in this table not always consistent with total sample size due to missing data on some items

### Qualitative feedback

Participants who took part in the CD interviews identified that this study "*is not trying to understand [diabetes] from a medical point of view but from a psychological point of view...because depression and diabetes...are best of friends*" (male, 25 years, type 1 diabetes). We found that people with diabetes were frustrated with the current health 'system' and that this survey asked questions which "*usually no one asks*" (female, 58 years, type 2 diabetes). The "*questions were quite probing...they scratched beneath the surface*" (male, 44 years, type 1 diabetes). One individual commented "*someone has finally done it...its needed.... there's so much frustration...within the diabetic system....when's it's all put together in voice and collected as a whole it's easy to get out there*"(male, 25 years, type 1 diabetes). Overall, Diabetes MILES--Australia has received a great deal of positive feedback from people with diabetes.

## Discussion

To our knowledge, Diabetes MILES--Australia is the largest national survey of the impact of living with diabetes ever performed in Australia; the largest in terms of sample size but also in terms of the breadth and depth of questioning. Diabetes MILES--Australia represents the first opportunity of its kind to assess the psychological health and unmet needs of a large and diverse sample of Australian adults with type 1 or type 2 diabetes. The dissemination of findings from Diabetes MILES--Australia over several years will raise awareness of the psychological and social impact of diabetes, as well as highlighting key associations between factors such as health status, health beliefs, social support, self-care, empowerment and a range of psychological outcomes. Findings will inform future research projects specific to the needs of Australians with diabetes and provide information that will assist in improving the capability of the NDSS to meet the needs of its registrants. Diabetes MILES--Australia, as described here, also represents the first step towards establishing a longitudinal program of research.

Diabetes MILES--Australia has been met with considerable enthusiasm by people with diabetes in Australia. Many participants commented on the significance of the study for them personally and for people with diabetes generally. Both the survey and the cognitive debriefing interview sessions enabled participants to "have a voice", expressing their thoughts, opinions, feelings and experiences of living with diabetes and healthcare provision.

The Diabetes MILES Study is now an international collaborative, with several colleagues making preparations to undertake similar initiatives in their own countries. Under the leadership of Prof Frans Pouwer of Tilburg University, the first to get underway is Diabetes MILES--The Netherlands, which completed its data collection in October 2011. Both datasets have included a core set of questions and measures (identified in Table 1) in order to have sufficient commonality of variables to enable meaningful international comparisons and pooling of data. Those interested in joining the Diabetes MILES Study International Collaborative are invited to contact Prof Jane Speight or Prof Frans Pouwer.

## Limitations

Surveying NDSS registrants who have consented to be contacted about research may not provide a fully representative sample of people with diabetes [54]. It is estimated that up to 50% of Australians with type 2 diabetes are undiagnosed, do not know they have the condition, and therefore are not on the register [55]. Registration information may include inaccurate or outdated details, such as diagnosis (e.g. type 1 when actually type 2 insulin-treated), treatment or demographics. The NDSS database is routinely cleansed, at least every six months, to correct or remove inaccurate age listings, addresses and update registrant deaths. However, in spite of this, 541 survey packages were returned to sender, because the addressee was either no longer residing at the address or was deceased.

Among the random sample of 15,000 registrants, 1,556 (10%) were listed as having carers. Two-thirds of carers (n = 1,023) were for registrants listed as having type 1 diabetes. In the instance where a carer was listed, the survey was automatically addressed to the carer, as is the default system on the NDSS database. In some instances, the carer listing was some years out of date. As such, some surveys may have been ignored, possibly lowering the response rate, simply due to the listed carer no longer being responsible for, living with, or in contact with the registrant.

Men account for 53% of NDSS adult registrants, and our sample included only 46% men. However, given that men are often more reluctant to participate in research (especially of a psychosocial nature), we were satisfied with this response. Further, although more than half of our sample were from just two of the eight Australian states and territories (namely New South Wales and Victoria), this reflects the proportion of NDSS registrants who reside in these states.

People of ATSI descent were under-represented in our sample, as were people of lower socioeconomic backgrounds, and people whose main language was not English. Diabetes MILES--Australia as it is described here was not designed specifically to meet the needs of people from culturally and linguistically diverse backgrounds, nor was it available in other languages, and so this result is unsurprising. Future Diabetes MILES--Australia initiatives may be tailored to meet the needs of these sub-populations to improve the accessibility of the study for these groups. However, reflective of the broader Australian population, a quarter of our sample indicated that they were born abroad.

The online survey was more likely to be completed by people with type 1 diabetes, women, younger adults, and those from Victoria or New South Wales. With the research team based in Victoria, there were more opportunities to advertise in Victorian media than in other states. Also, there were more opportunities to advertise in media aimed at adults with type 1 diabetes. Although older adults had lower rates of participation in the online survey than their younger counterparts, more than 20% of the online survey sample was aged over 55 years, and those who took part spanned the whole eligible age range (18-70 years), suggesting that online research is not inaccessible to older adults.

## Conclusions

Diabetes MILES--Australia represents a major achievement in the study of diabetes in Australia, where there has been limited published research on the psychosocial and behavioural aspects of this condition. For the first time, an in-depth study has been conducted at a national level, which has the potential to provide much needed data on a range of important psychological outcomes hitherto neglected. Diabetes MILES--Australia also represents the baseline of a longitudinal cohort study, which will enable prospective investigation of psychological outcomes and how they relate to the natural history of diabetes, its treatment and the onset of complications and other co-morbidities.

## Endnotes

^a ^State-based data accurate as of 30 June 2011. ^b ^Gender-based data accurate at December 2010.

## Abbreviations

ANOVA: Analysis of variance; ATSI: Aboriginal or torres strait islander; CD: Cognitive debriefing; CRO: Contract research organisation; DAWN: Diabetes attitudes wishes and needs study; MILES: Management and impact for long-term empowerment and success; NDSS: National diabetes services scheme; SPSS: Statistical package for the social sciences.

## Competing interests

The authors declare that they have no competing interests.

## Authors' contributions

JS conceived The Diabetes MILES Study, and together with FP developed The Diabetes MILES Study International Collaborative. JS is principal investigator of Diabetes MILES--Australia; she developed the proposal and designed the study; she took overall responsibility for the development of survey content, and supervision of the research team; she produced the first draft of this manuscript. JB contributed to the development of survey content, conducted data cleaning and analyses, and contributed substantially to the development of this manuscript. EHT was the project manager; she conducted the literature searches, liaised with reference group members, conducted CD interviews, contributed to the development of survey content, managed the survey data collection, and conducted data cleaning and analyses. CH provided expert advice throughout the data cleaning process and contributed substantially to the development of this manuscript. All authors commented on the initial draft and approved the final manuscript.

## Pre-publication history

The pre-publication history for this paper can be accessed here:

http://www.biomedcentral.com/1471-2458/12/120/prepub
